# SUMOtherapeutics for Ischemic Stroke

**DOI:** 10.3390/ph16050673

**Published:** 2023-04-29

**Authors:** Paramesh Karandikar, Jakob V. E. Gerstl, Ari D. Kappel, Sae-Yeon Won, Daniel Dubinski, Monica Emili Garcia-Segura, Florian A. Gessler, Alfred Pokmeng See, Luca Peruzzotti-Jametti, Joshua D. Bernstock

**Affiliations:** 1 T. H. Chan School of Medicine, University of Massachusetts, Worcester, MA 01655, USA; 2 Department of Neurosurgery, Brigham and Women’s Hospital, Harvard Medical School, Boston, MA 02215, USA; 3 Department of Neurosurgery, Boston Children’s Hospital, Boston, MA 02215, USA; 4 Department of Neurosurgery, University Medicine Rostock, 18057 Rostock, Germany; 5 Department of Clinical Neurosciences, University of Cambridge, Cambridge CB2 0QQ, UK; 6 NIHR Biomedical Research Centre, University of Cambridge, Cambridge CB2 0QQ, UK; 7 Koch Institute for Integrated Cancer Research, MIT, Cambridge, MA 02142, USA

**Keywords:** stroke, ischemia, neuroprotection, SUMOylation, experimental therapeutics

## Abstract

The small, ubiquitin-like modifier (SUMO) is a post-translational modifier with a profound influence on several key biological processes, including the mammalian stress response. Of particular interest are its neuroprotective effects, first recognized in the 13-lined ground squirrel (*Ictidomys tridecemlineatus*), in the context of hibernation torpor. Although the full scope of the SUMO pathway is yet to be elucidated, observations of its importance in managing neuronal responses to ischemia, maintaining ion gradients, and the preconditioning of neural stem cells make it a promising therapeutic target for acute cerebral ischemia. Recent advances in high-throughput screening have enabled the identification of small molecules that can upregulate SUMOylation, some of which have been validated in pertinent preclinical models of cerebral ischemia. Accordingly, the present review aims to summarize current knowledge and highlight the translational potential of the SUMOylation pathway in brain ischemia.

## 1. Introduction

Despite landmark developments in chemical thrombolysis and mechanical thrombectomy in the past decades, ischemic stroke remains one of the greatest drivers of global disease burdens [1,2,3,4]. Effective management options are often not available due to the temporality of presentation and the lack of access to specialist medical facilities, even in high income settings. Eligibility for alteplase/tenecteplase is dependent on patient presentation within 4.5–9 h and the absence of disqualifying comorbidities [5]. Endovascular thrombectomy is an effective method for revascularization but is not available to many patients, as they are either not candidates for intervention, do not have access to a skilled operator at a stroke-capable center, or may not be present within the time window for intervention. In fact, a significant fraction of patients with ischemic stroke do not benefit from rapid recanalization—for example, between 2012–2018 in the United States, only 3.5% of stroke patients received a thrombectomy, 9.3% received tPA, and 1% received both [6]. While the positive results reported in the DAWN and DEFUSE-3 trials have likely spurred the wider utilization of these methods, the fact remains that the treatment paradigm remains the same: existing care for stroke focuses on the physiologic effects of flow and perfusion on bulk tissue and does not account for the cellular or molecular biological mechanisms of ischemic stroke. Even with advances in imaging to identify patients with less tissue injury and persistent at-risk tissue, about half of patients taken for stroke intervention still have unfavorable outcomes (54% [7], 56% [2], and 51% [3]). On the other hand, RESCUE-Japan LIMIT [8], SELECT2 [9], and ANGEL-ASPECT [10] show that patients with significant tissue injury can still benefit from reperfusion.

Although structural vessel occlusion is the initiating event in clinical stroke, the pathobiological sequelae that unfold as a result of such ischemia must ultimately also be targeted, and as such, the expansion of treatment paradigms to include novel neuroprotective therapies will be required both for patients that are successfully treated (e.g., subjective to reperfusion injury) and those that are not. Accordingly, herein, we provide a clinically oriented summary of the current understanding of the role of the small, ubiquitin-like modifier (SUMO) protein in endogenous neuroprotective mechanisms, recapitulate known therapeutic candidates acting on SUMO pathways (SUMOtherapeutics), and conclude by exploring potential future directions and approaches within the context of ischemic stroke.

### 1.1. Biological Significance of the SUMO Pathway

SUMO is a protein intrinsically involved in orchestrating physiological responses to hypoxia, hypothermia, and DNA damage [11]. The SUMO conjugation of proteins (SUMOylation) was first implicated in the torpor conditioning of the 13-lined ground squirrel (*Ictidomys tridecemlineatus*) [12]. The analogous nature of this physiological mechanism of hibernation to the pathological mechanism of ischemic stroke and arousal to reperfusion has made it a direction for inquiry in the context of neuroprotection [12,13,14]. The postmortem observations of increased SUMOylation within the penumbra of ischemic stroke in human victims has made the potential exploitation of this pathway of clinical interest [15]. 

The SUMO pathway exerts its effects by way of post-translation modification akin to ubiquitinylation. However, while only 29 variants of ubiquitin-binding domains have been identified in humans at the time of writing, over 14,000 SUMO binding domains have been found in human cells [16]. Conversely, whereas ubiquitinylation involves one of almost three dozen forms of the E2 conjugase, SUMOylation relies solely on one—UBC9 [17]. This combination of a vast number of SUMO-binding domains coupled with a universal reliance on a single E2 conjugase has significant potential as a wide-reaching yet approachable therapeutic target [17,18].

Although four SUMO paralogs have been identified in humans, SUMO-1 and SUMO-2/3 (SUMO-2 and SUMO-3 being almost identical) represent the prevalent isotypes [17,18]. The act of conjugating/deconjugating SUMO, SUMOylation, and de-SUMOylation, is primarily carried out by seven Sentrin-specific proteases known as SENP1-7. Of this family, SENP1 (key for maturing translated SUMO) and SENP2 display the broadest substrate affinity for SUMO isotypes and have been found preferentially within the nucleus and nuclear pore complex. The other SENPs are either selectively active on a particular SUMO isoform or are not involved in SUMO maturation, making them of lesser interest for SUMOtherapeutics engineered for global upregulation. Other SUMO-involved enzymes, such as DeSI-1, DeSI-2, and USPL1, have been identified but have been found to have weak endopeptidase activity and are not thought to be involved in global SUMOylation to the extent of SENP1 and SENP2 [11,19,20].

The exact extent of control exerted by SUMO and the bounds of its physiological interplay remain to be fully elucidated. SUMOylation is involved in numerous processes spanning most organ systems, with downstream effects occasionally resulting in the simultaneous upregulation and downregulation of protein machinery, such as HIF-1α [11]. Knockout experiments have demonstrated that SUMO has a role, albeit undefined, in emotion, cognition, anxiety, and episodic memory [21,22]. Preclinical work has shown that SUMO serves as a regulator of critical cellular processes, including DNA repair, axonal trafficking, and brain development, as well as neurotransmission and neuroplasticity [19,20,23,24,25,26]. Neuroplasticity may represent another possible context for therapeutic SUMO-modulation. SUMOylation has, for example, been identified as a critical coordinator of both pre- and post-synaptic architecture in synapse formation [27,28]. The interested reader is directed to an excellent review by Luo et al. [27]. 

The importance of SUMOylation has also been observed in disease states: SUMOylation and de-SUMOylation have been identified as potential drivers of various CNS pathologies. Glioblastomas have been found to overexpress SUMO [22]; increased SUMOylation has been associated with Alzheimer’s Disease and Huntington’s Disease; and both increased SUMO1ylation and decreased SUMO2/3ylation have been implicated with α-synuclein aggregation in Parkinson’s disease [29]. Moreover, SUMOylation is known to play a role in cardiac [30,31], renal [32], pulmonary, hepatic, and mesenteric ischemic disease processes [14]. These observations, coupled with reports on the critical role of SUMO in cell survival during hyperacute ischemic pathologies, such as myocardial infarction [33], make it of particular interest for the management of acute ischemic stroke.

### 1.2. Role of SUMO in Neuroprotection

The neuroprotective effects of SUMOylation have been widely observed both in vitro and in vivo [14]. Due to the role of SUMO as the “modifier of modifiers” and the great physiological distance between the act of SUMOylation and its ultimate effect; a comprehensive pattern of association between end-effects and neuroprotection remains to be clearly demonstrated [14]. The importance of SUMO in preserving neuronal integrity first observed in natura has been replicated in vitro and in vivo through key preclinical work [19,21,23,25,26,34]. Furthermore, recent proteomics-based approaches continue to produce new mechanistic insights into the downstream effects of SUMO in contexts such as the response to cellular stress, development, and differentiation. Despite that, a clear and comprehensive awareness of causal relationships is yet to be defined.

The preclinical literature offers a variety of downstream effects of SUMOylation that may explain the observed impacts [35]. As with other advances in systems biology, the greater awareness of the panoply of interactions governed by SUMO represents an expanding list of potentially druggable nodes [35]. A large portion of studies to date have demonstrated the importance of both SUMO1 [36] and SUMO2/3 [34] in neuroprotection. As SUMO proteases play a central role in neuroprotection, factors regulating their activity should be considered. For example, SENP1 and 3 have been associated with the reversible modulation of activity dependent on levels of reactive oxygen species or genotoxic stress, suggesting a possible role as intracellular sentinels for impending insult [20,37]. The interested reader is directed to excellent reviews by Droescher et al. and Filippopoulou et al., covering the variety of both the physiologic and protective roles SUMO plays within the cell [11,20].

#### 1.2.1. SUMO and the Ischemia Response

The neuroprotective effects of SUMO regulation are most apparent in the context of its response to physiologic stressors, including hypoxic and ischemic insults [38]. As with other aspects of SUMO-related research, the complete breadth of control exerted by SUMO over the ischemic stress response remains to be comprehensively defined [38]. There are increasing reports of SUMO-interactions with first- and second-order stress pathways integral to neuronal survival. As such, SUMO is believed to be a critical intermediary in optimizing and balancing the various endogenous stress responses to favor regeneration and repair as opposed to apoptosis and degeneration.

The hypoxia-inducible factor (HIF) is a family of transcription modifier proteins that play a central role in hypoxic response, wound-healing, and angiogenesis. In the context of ischemic stroke, HIF activation is associated with increased protection and recovery after insult [39]. Although HIF expression varies based on ambient oxygen tension and interactions with other pathways, such as Nuclear Factor κB (NF-κB), prolonged activation has been associated with non-scarring tissue regeneration and is a therapeutic target for several other pathologies [40]. Unsurprisingly, both SUMOylation and the SUMO pathway enzymes have been observed to play a role in both the positive and negative regulation of the HIF pathway, with varying effects depending on both the agent and the target of SUMOylation [11]. The differing effects of the family of SUMO-associated E3 ligases is an example of the former—depending on which ligase is involved, HIF transcription may be positively regulated, negatively regulated, both, or unaffected. The E3 ligases Cbx4 and PIAS3 positively impact HIF-1α stability and transcription with and without SUMOylation, respectively [41,42,43]. Alternatively, PIAS1 SUMOylates and inhibits HIF-1β (encoded by *ARNT*) without an appreciable effect on overall HIF transcription [44]. In some cases, opposing effects can be produced by the same E3 ligase, depending on the target of SUMOylation—PIASγ SUMOylation of HIF-1α negatively impacts HIF stability and transcription, but PIASγ SUMOylation of pVHL results in the increased ubiquitinylation of the same, ultimately resulting in increased HIF stability [45,46]. Similar modulating SUMO interactions with the prolyl and asparaginyl hydroxylases that serve as negative feedback to HIF activation have also been reported: the SUMOylation of PHD3 and FIH results in downregulation (via the potentiation of extant negative feedback) and the upregulation of HIF transcription [47,48]. Finally, experiments in HeLa cultures subjected to oxygen–glucose deprivation (OGD) have demonstrated that changes in SUMOylation in more generalized transcription factors can inflect the activity of HIF-1—specifically, a reduction in the SUMO2/3ylation of TFAP2 results in an increase in the transcriptional activity of the same while also enhancing that of HIF-1 [49].

Whereas the activation of HIF-1 is associated with neuroprotection, the upregulation of NF-κB has been observed to correspond with inflammation and neuronal death [50]. Similar to its impact on HIF, SUMO pathways affect multiple nodes within the NF-κB pathway, occasionally with contradicting impacts. The known SUMO influences within the pathway relate primarily to the IκBα and the IKK complex [40]. IκBα is a constitutively active inhibitor that functions by preventing the nuclear localization of NF-κB. Experiments with over-expressed dominant negative murine homologues of Ubc9 observed delays in IκBα degradation and thus the activation of the pathway after in vitro exposure to TNFα, implicating the SUMO E2 in the endogenous activation of NF-κB [51]. At the same time, SUMO1ylated IκBα was discovered in COS7 monkey kidneys, HEK-293, and Jurkat human T lymphocyte cultures and was found to be resistant against ubiquitinoylation. Furthermore, the same study found that ubiquitin and SUMO1 competed for the same lysine residue (K21) within IκBα, suggesting competing influences of SUMO and Ubiquitin on IκBα stability and NF-κB activation [40,52]. Similarly, SUMO has been found to exert indirect influence over IκBα by way of the IKK complex. IKK serves to enable the nuclear localization of NF-κB by phosphorylating IκBα. The SUMOylation of Annexin-A1 was found to suppress the NF-κB pathway by increasing IKKα degradation in a microglial model of oxygen–glucose deprivation [53]. On the other hand, the SUMO1ylation of IKK-γ (also known as NEMO) resulted in greater nuclear trafficking and thus the activation of NF-κB [54]. The proinflammatory impact of SUMO1ylating NEMO was demonstrated in reverse via the observation that the in vitro overexpression of SENP1 resulted in increased de-SUMOylation of NEMO and concomitant reduction in NF-κB activation [55].

In addition to HIF-1 and NF-κB, SUMO has been found to act as a modulator, albeit less characterized, of many other cellular pathways. STAT, a signaling pathway involved in the inflammatory response, was suppressed by the SUMOylation of synthetic liver X receptors (LXR) [56]. The SUMOylation of the GluR6 subunit of the Kaianate Receptor (KAR) has been observed to downregulate the JNK cell-death pathway. Similarly, the increased SUMOylation of the GluR2 subunit of the AMPA receptor has been observed in a murine model of middle cerebral artery occlusion [57]. Finally, the OGD-induced degradation of SENP3 by PERK has been found to result in the increased SUMO2/3ylation of Drp1, resulting in greater survival during ischemia but increased death during reperfusion [58,59,60]. As such, SUMO is integrally linked with the physiological response to ischemia.

#### 1.2.2. Maintenance of Ion Homeostasis 

Ion homeostasis is a critical factor in neuronal survival following an ischemic insult. SUMOylation directly or indirectly impacts the balance of calcium, sodium, and potassium within the neuron [59]. Hypoxic-ischemic injury to neurons is known to lead to the massive disruption of Ca^2+^ and Na^+^ homeostasis, uncontrolled neurotransmitter release, and subsequent excitotoxicity [61]. Artificial increases in synaptosomal SUMO1 and SENP1 have been shown to modulate calcium intake and glutamate release with opposing results observed with Kainate or KCl stimuli [28,62]. In neurons of the dorsal root ganglia, increased SUMOylation has been found to reduce calcium influx and increase the membrane migration of sodium channels [63]. The impact of SUMOylation on calcium homeostasis is thought to be due to regulatory interplay with the NCX3 sodium–calcium exchanger at the LYS590 f-loop: experiments with the siRNA-induced knockdown of SUMO1 demonstrated a corresponding downregulation of NCX3 and thus the exacerbation of ischemic damage in a rat model of MCAO [36].

In addition to the direct impact on ion transporters themselves, SUMOylation has been observed modulating receptor expression, possibly as a means of mitigating excitotoxicity and thereby blunting the aforementioned calcium influx. The SUMOylation of the GluR6 subunit of the Kaianate receptor (KAR) has been found to cause a reduction in end-synaptic potential [64]. In line with these observations, SUMOylation has been shown to modulate synapse formation, neurotransmitter release via SNARE interactions, and synaptic plasticity, suggesting a fundamental role within neuronal synaptic architecture [65].

#### 1.2.3. Preservation of Neural Stem Cell Populations 

The brain maintains a limited ability to repair and regenerate itself by way of its small host of native neural stem cells (NSCs). However, its inability to fully compensate for the overwhelming damage caused by ischemic insults is evidenced by the profound morbidity that may follow ischemic stroke [6,66]. Furthermore, advances in regenerative medicine brought about by exogenous stem cell therapies in other contexts are yet to be replicated for neuropathological indications [67,68]. Preclinical successes have been observed with graft preconditioning and the use of scaffolding, but there are considerable challenges associated with such clinical translation [58].

The SUMO pathway also plays a critical role in the aforementioned quotidian homeostatic processes and responses to stress [58]. Moreover, its role in cellular development and differentiation has been established by the discovery that the SUMOylation of chromatin is integral to the maintenance of cellular identity—particularly that of the pluripotent stem cell [17]. Tahmasebi et al. reported not only an increase in Ubc9 expression during the transformation of murine embryonic fibroblasts into induced pluripotent stem cells (iPSCs) but also a profound decrease in iPSC transformation when Ubc9 functionality was exogenously suppressed [69]. Indeed, murine experiments found that the knockout of either SUMO (in particular SUMO2) or associated proteases were lethal in utero [17,70]. In this way, SUMOylation may represent a means of facilitating the survival and possibly the expansion of endogenous NSC populations.

In the context of ischemic insult, the use of SUMOylation to prepare exogenous NSCs for infarct-like conditions may serve as a form of in vitro preconditioning to enhance the chances of exogenous graft survival and expansion [66]. This concept has been demonstrated both in vitro and in vivo: cultured NSCs with induced SUMO upregulation were found to have greater survivability against OGD and increased differentiation both within and without the murine brain [71]. Although the technology remains in its infancy, it has the potential to cause a paradigm shift in ischemic stroke—from damage control to anatomical and functional repair.

## 2. SUMOtherapeutics for Cerebral Ischemia

**Figure 1:**
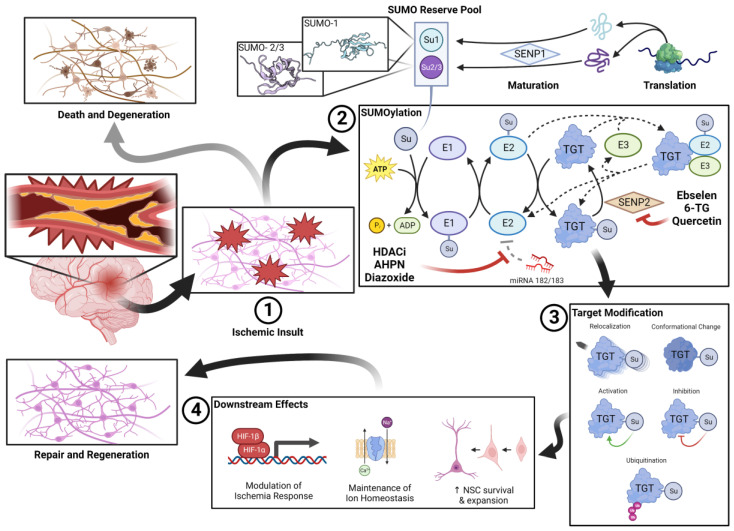
SUMOylation in cerebral ischemia. A pool of unconjugated SUMO is kept in reserve during normoxic conditions. After vascular occlusion, (1) the lack of perfusion results in an ischemic insult. When stressors are detected within the microenvironment, the conjugation of the SUMO reserve is rapidly upregulated. SUMOylation (2) is a process analogous to that of ubiquitination. The process of SUMOylation and de-SUMOylation is highly dynamic and the subject of significant regulatory interplay. Endogenous deSUMOylators, such as SENP2 and miRNA-182/183, thus represent therapeutic targets. SUMOylated target proteins have a variety of fates (3) that may impact their functionality, conformation, or location within the cellular milieu. Ultimately, SUMOylated proteins affect a variety of (4) pro-survival and pro-regeneration changes, including managing the response to ischemia, managing ion homeostasis, and encouraging the survival of endogenous/exogenous neural stem cell (NSC) populations. To this end, the regenerative nature of the SUMO response to ischemia stands in stark contrast against the apoptotic and degenerative response that is prevalent within the ischemic brain.

As the awareness of SUMOs regulatory function expands, efforts to identify potentially druggable nodes and small molecule agents to exploit this are already underway [35]. These SUMOtherapeutics can be broadly classified as either targeting a step within the SUMO pathways or as a direct upregulator of SUMOylation (Figure 1) [35]. The former class is unsurprisingly composed of compounds antagonizing endogenous SUMO inhibitors, such as miRNA-182/183, and inhibiting deSUMOylating proteases, such as SENP-2. Many of these so-called “inhibitor–inhibitors” have been identified through novel screening assays and are currently undergoing evaluation.

### 2.1. miRNA-182/183 Inhibitors 

Micro-RNA is a negative regulation system that functions by silencing or degrading target mRNA. Based on experiments on hibernating ground squirrels, two major miRNA families have been identified in global SUMO suppression—miRNA-182 (including miR-182, miR-183, and miR-96) and miRNA-200 (including miR-200a/b/c, miR-141, miR-429) [72,73]. As such, therapeutic upregulation and downregulation are simultaneously of interest due to the implication of SUMO in other pathologies [35,74]. Advances in quantitative high-throughput screening have enabled the identification of miRNA-182 antagonists with druggable properties [75] (Table 1).

Despite many of the drugs found through screening being in the preclinical stages of investigation, the histone deacetylase inhibitors (HDACi) romidepsin, panabinostat, entinostat, belinostat, and pracinostat are either currently clinically utilized or part of ongoing clinical evaluation [75]. Panabinostat is of particular interest, as it was found to be one of the most effective miRNA inhibitors when evaluated for both SUMO upregulation and protection against OGD [75]. Given that HDAC activity has been positively correlated with an increased penumbra diameter in recent transcriptomics studies [76] and the encouraging in vivo performance of other HDACi (albeit ones with unknown interactions with SUMO) in models of stroke [77,78,79], the frequency of HDACi amongst the candidates identified during screening is of interest. However, the in vivo evaluations of panabinostat and entinostat have been mixed; a study by Al Shoyaib et al., studying the effects of both drugs (oral formulations, dosed on alternate days from post-stroke day 5 to 15) on motor recovery in a photothrombotic model of ischemic stroke in CD-1 mice, did not find statistically significant differences in motor recovery or infarct volume when compared to vehicle [80]. Another study in a CD-1 mouse model of intracerebral hemorrhage by Bonsack and Sukumari-Ramesh evaluated the effect of entinostat (delivered intraperitoneally 1 h after insult at 10mg/kg in PBS) on sensorimotor deficit, neuroinflammation, acute neurodegeneration, and lesion volume and found significant reductions in all measures compared to control [81]. It is important to note that the relative paucity of other in vivo data on these specific drugs for this indication and extremely varied experimental parameters of the aforementioned studies makes comparing the results challenging.

The selective mitochondrial potassium-channel agonist diazoxide has also been identified as an inhibitor of miRNA-182/183. Observations of its protective effects on myocardium in ischemic heart disease inspired studies on its use in cerebral ischemia. A study evaluating infarct volume in a Wistar rat model of middle cerebral artery occlusion (MCAO) found an over two-fold reduction after diazoxide pretreatment [82]. Another study measured the cerebral concentrations of heat-shock protein 25 and 70 (HSP25, HSP70) in the brains of Sprague Dawley rats subject to cerebral ischemia as a consequence of hemorrhagic shock and observed significant increases in concentration through both pretreatment and posttreatment with diazoxide [83].

**Table 1:** miRNA-182/183 inhibitors evaluated for cerebral ischemia.

Drug	Tissue/ Animal	Ischemia Model	Intervention	Measured Outcome	Results Summary	Study
Orotic Acid	SHSY5Y, E18 PCN	SHSY5Y: 15 h OGD + 6 h recoveryE18 PCN: 5 h OGD + 16 h recovery	Drug co-treatment	Cell survival, SUMO concentration	SUMO upregulation *, OGD protection *	Bernstock et al., 2016 [75]
AHPN						
Telmisartan						
TW-37						
Dianiline						
Diazoxide						
NCGC00185916					SUMO upregulation *	
Romidepsin						
VX-702						
Lenalidomide						
Belinostat						
Pracinostat						
Licofelone						
Fosmidomycin						
JWH-015						
Motesanib						
Vatalanib						
Entinostat						
Panobinostat						
Entinostat	CD-1 mice	Photothrombotic stroke at PMCA	Drug post-treatment (post stroke day 5–15)	Motor recovery, infarct volumes	No difference vs. negative control for both measures	Al Shoyaib et al., 2016 [80]
		Collagenase-induced ICH	Drug post-treatment (1 h post stroke, 10 mg/kg IP in PBS)	Sensorimotor deficit score, CD16/32 expression, neurodegeneration (via TUNEL staining neurons), infarct volume	Reduction in day 1 and day 3 post-ICH sensorimotor deficit *. Reduction in CD16/32 expression *, neurodegeneration, and infarct volume *	Bonsack and Sukumari-Ramesh, 2021 [81]
Diazoxide	Wistar rats	1.5 h MCAO	Drug pretreatment (15 min prior to stroke, 30 μL 0.4 mM or 2 mM ICV bolus)	24 h post-stroke neurological score, infarct volume	Increase in neurological score *; reduction in infarct volume *	Shimizu et al., 2002 [82]
	SD rats	1 h RCCA ligation + hemorrhagic shock	Drug pre- and post-treatment: 5 mg/kg IP bolus 24 h pre-stroke; 2.3 mg/kg/10 min IV infusion 10 min or 60 min posttreatment	HSP25 and 70 concentrations	Pretreatment: upregulation of HSP25 and 70 *; 60 min posttreatment: upregulation of HSP25 and 70 *	O’Sullivan et al., 2007 [83]

* Results are statistically significant. Abbreviations: HSP, heat shock protein; ICH, intracerebral hemorrhage; IP, intraperitoneal; MCAO, middle cerebral artery occlusion; OGD, oxygen–glucose deprivation; PCN, primary cortical neuron; PMCA, primary motor cortical area; RCCA, right common carotid artery; SD, Sprague Dawley.

### 2.2. SENP Inhibitors

In addition to the significant research effort that has been directed towards the understanding of the physiological role of SUMO, other efforts have focused on identifying potential therapeutic candidates by way of recent advances in high-throughput screening [84]. Bernstock et al. developed AlphaScreen (PerkinElmer, Waltham, MA, USA) as a facile and efficient means of identifying SENP-2 inhibitors (measured by an overall increase in SUMOylation). The primary screening batch consisted of over four thousand compounds from the Sigma LOPAC^1280^ (Library of Pharmaceutically Active Compounds) and NCATS NPC (National Center for Advancing Translational Science Pharmaceutical Collection), of which less than two hundred remained after screening. After further in vitro and in vivo testing, eight drugs were identified as lead candidates, of which a final four significantly increased SUMO conjugation (Table 2) [84]. Of these candidates, antioxidant ethyl protocatechuate and nonselective beta antagonist isoprenaline HCl have not been studied in the context of stroke. The nucleoside analogue 6-Thioguanine has also not been evaluated in the context of stroke, but its parent nucleoside has been identified as a SUMOylator and neuroprotectant [85].

Quercetin, a flavonoid commonly found in citrus fruits, onions, and broccoli, is a natural antioxidant found to have SENP-inhibiting properties [86]. Although successes in stroke prevention or neuroprotection enhancement have been widely reported in the preclinical literature, no consensus exists on the mechanism of action. The preclinical literature suggests several possible mechanisms, including the reduction of thrombotic inflammation, antithrombotic effects, the prevention of oxidative stress, the promotion of autophagy, and the inhibition of apoptosis [87]. Although a recent meta-analysis of in vivo studies reported the neuroprotective effects of quercetin for ischemic stroke, it found evidence of publication bias and methodological issues, such as a lack of blinding [88]. At the time of writing, there has been no clinical advancement of quercetin as a therapeutic.

As another example of a known neuroprotectant that remains mired in translation, ebselen is a selenium-containing glutathione peroxidase mimic that was a known neuroprotective agent even before its SUMOylating properties were discovered. Furthermore, at least one mechanism for its neuroprotective properties other than its capacity as a SUMOylator has been elucidated—it is able to significantly mitigate the deleterious effects of glutamate excitotoxicity [89] and reperfusion injury [90]. At the turn of the 21st century, it was a promising candidate for a putative protective agent for stroke, going so far as entering clinical trials in Japan. Although trial data were encouraging, showing statistically significant improvements in infarct volume and functional status [91,92], these trial data did not approach the efficacy observed in animal models and in vitro studies. This, coupled with concerns over selenium toxicity caused regulatory approval and clinical implementation to stall [93,94].

As such, the corpus of small animal and tissue culture research on ebselen and quercetin depicts a pattern of encouraging results hampered by difficulties in clinical translation and a lack of standardization and may merit revisiting. For example, clinical trials utilized oral suspensions of ebselen, significantly increasing off-target exposure due to its lipophilic nature. Recent advances in catheter technology and mechanical thrombectomy could enable the precise and controlled dosing of the compound [95,96]. Such an approach would have the benefit of limiting off-target exposure and enabling dosing as soon as the thrombus is retrieved and reperfusion injury begins to occur—a characteristic correlated with increased neuronal survival [95,96].

In addition to revisiting older compounds, such as ebselen or quercetin, with recently discovered SENP inhibitor characteristics, the potential for design and discovery of novel agents is increasingly at play. With recent advances in high-throughput screening (such as the previously mentioned AlphaScreen), AI-assisted drug design, and other in silico methods, investigating SENP inhibition is of great interest in a variety of clinical contexts. The interested reader is directed to an excellent review by Chen et al. for further information about the development of SENP inhibitors for a variety of other clinical indications [97]. 

**Table 2:** SENP inhibitors evaluated for neuroprotection.

Drug	Tissue/ Animal	Ischemia Model	Intervention	Measured Outcome	Results Summary	Study
Quercetin	SHSY5Y, B35, E18 PCN	SHSY5Y, B35: 16 h OGD + 5 h recovery E18 PCN: 5 h OGD + 16 h recovery	Drug treatment ± pre-treatment	SENP activity, SUMOylation, cell survival, LDH release	Decrease in SENP expression *; increase in SUMOylation *; increase in cell survival (in SHSY5Y and E18 PCN) *; decrease in LDH release with co-treatment alone and with pre-treatment *	Lee et al., 2016 [86]
Isoprenaline HCl	B35	20 h OGD	4 h pretreatment + treatment	SUMO-1 expression, cell survival	Increase in SUMO-2/3 conjugation *, no significant OGD protection	Bernstock et al., 2018 [84]
Ethyl protocatechuate					SUMO-1 upregulation *, OGD protection *	
6-thioguanine	C57BL6 mice	N/A	12 mg/kg IP bolus treatment	SUMOylation 1 h after bolus	Increases in SUMO-1 and SUMO-2/3 conjugation *	
Ebselen	SHRSP, WKY rat PDN	24 h OGD + 3 h recovery	treatment + 3 h posttreatment	Cell survival, LDH activity	OGD protection *, no significant difference in LDH activity	Yamagata et al., 2008 [98]
	SHRSP, WKY rat	0.5 h BCCO	30 mg/kg/day pretreatment for 7 days, then 30 mg/kg/day posttreatment for 3 days	Apoptotic neurons in CA1 subfield of hippocampus	Almost complete inhibition of apoptosis ^†^	
		N/A	60 mg/kg/day treatment for 6 weeks	Oxidative stress (via cortical NO and MDA concentrations); iNOS expression	Reduction in NO and MDA concentrations *; reduction in iNOS expression *	Sui et al., 2005 [99]
	Human	MCAO	150 mg PO BID post-treatment within 12 h of onset for 2 weeks. Placebo controlled, double blind trial.	Infarct volume 1 mo post stroke, GOS 3 mo post stroke	Reduction in infarct volume *; superior GOS if administered within 6 h *; no significant difference in GOS vs. negative control overall	Ogawa et al., 1999 [91]
		AIS	150 mg PO BID posttreatment within 48 h of onset for 2 weeks. Placebo controlled, double blind trial.	GOS (1- and 3-month), neurological status (2 weeks, modified Mathew Scale), functional status (2 weeks, Barthel Index)	Improvement in 1-month GOS * but no significant difference in 3-month GOS; superior GOS if administered ≤24 h *; reduction in both impairment (Mathew) * and disability (Barthel) *	Yamaguchi et al., 1998 [92]
	SD rats	Permanent MCAO	1 mg/kg/h pretreatment 45 min pre-stroke to 4 h post-stroke	Extent of ischemic damage, oxidative stress (via IHC)	28% reduction in cortical ischemic damage vs. control ^†^; reduction in oxidative stress markers vs. control ^†^	Imai et al., 2003 [100]
		2 h FCI	1 mg/kg IV bolus + 1 mg/kg/h IV post-treatment for 24 h	24 h neurological deficit, gray matter damage, Axonal damage, and oxidative stress (via IHC)	40.7% reduced neurological deficit at 24 h vs. control *; 53.6% reduction in gray matter damage *; 46.8% reduction in axonal damage IHC markers *	Imai et al., 2001 [101]
	Wistar rats	45 min BCCO	30 mg/kg PO bolus pretreatment 2 h prior to stroke	cortical EAA and NO concentrations, 24 h hippocampal CA1 subfield integrity	Increase in intact CA1 neurons *; no difference in EAA or NO concentrations vs. control.	Koizumi et al., 2011 [89]
		2 h FCI	10 mg/kg and 100 mg/kg PO bolus pretreatment 1 h prior to FCI	Reduced glutathione concentration; plasma Selenium; 1 week post stroke infarct size	Increase in perfusion with 100 mg/kg *; increase in plasma Selenium *	Salom et al., 2004 [102]
	Wistar rat cerebellar neurons	Glutamate exposure	25 min treatment ± posttreatment; posttreatment	24 h cell survival, 48 h cell survival	Increase in survival with treatment * and posttreatment * comparable to negative control	Porciúncula et al., 2001 [95]
	Wistar rat hippocampal neurons	45 min OGD	Pretreatment and posttreatment	3 h cell survival	Increase in survival with treatment * and post-treatment * comparable to negative control	Porciúncula et al., 2003 [96]

* Results are statistically significant. ^†^ No measurement of statistical significance presented. Abbreviations: AIS, acute ischemic stroke; BCCO, bilateral common carotid occlusion; EAA, excitatory amino acid; FCI, focal cerebral ischemia; GOS, Glasgow outcome scale; IHC, immunohistochemistry; iNOS, induced nitric oxide synthase; MCAO, middle cerebral artery occlusion; MDA, malonaldehyde; NO, nitrous oxide; OGD, oxygen–glucose deprivation; PCN, primary cortical neuron; PDN, primary dissociated neuron; PO, per os; SD, Sprague Dawley; WKY, Wistar Kyoto.

### 2.3. Direct SUMO Upregulators

The increased accessibility and prevalence of high-throughput screening methodologies has enabled the rapid development of libraries of possible SUMOtherapeutic agents. Krajnak et al. and Neurodon LLC (Schererville, IN, USA) utilized a Förster resonance energy transfer (FRET)-based high-throughput screening tool to identify prospective SUMOylators, which were further screened in orthogonal assays and filtered based on physicochemical criteria indicating druggability. While the exact compounds utilized remain proprietary, the authors identified eleven small-molecule SUMOylators belonging to the quinoline, benzothiazole, and aminothiazole families. When evaluated in an in vitro model of endoplasmic reticulum stress (via thapsigargin exposure) in CSM14.1 striatal neuroprogenitor and Buffalo Green Monkey Kidney cells, nine out of the eleven identified compounds demonstrated statistically significant SUMO upregulation compared to control [103]. Despite these compounds being developed for clinical indications other than ischemic stroke, the relatively facile nature of such screening tools represents a promising avenue towards an ever-expanding library of SUMOylators. 

## 3. Near Future Innovation in SUMOtherapeutics

The expanding understanding of the SUMO pathway is generating an increasing number of potential therapeutic indications [104]. In turn, compounds that modulate the pathway are increasingly the subject of research efforts for a variety of conditions, including ischemic diseases of the heart, kidney, and transplanted organs [16,104]. With viable solutions to the problems of substrate availability [105] and screening methods [84], the preclinical investigation of the SUMO pathway is increasingly facile. In fact, the first clinical trials of a SUMOtherapeutic compound, TAK-981 (Takeda Pharmaceuticals, Cambridge, MA, USA), are ongoing at the time of writing (NCT03648372, NCT04074330, and NCT04381650) [106]. While it is a chemotherapeutic agent, the lessons learned during its development will undoubtedly aid in the development of other SUMOtherapeutics.

Due to the gross similarities between the SUMO pathway and ubiquitinoylation, it is not unreasonable that the development of SUMOtherapeutics would follow the trajectory of the latter’s therapeutic exploitation. Advances in high throughput screening and in proteomics methods along with the greater availability of equipment enable SUMOylation research to be conducted at an increased pace. Unfortunately, unlike agents targeted to other indications, SUMOtherapeutics for cerebral ischemia remain closer to the bench than the bedside.

Even so, in a new ‘era of neuroprotection’, advancements in other therapeutic technologies may represent a catalyst for stroke SUMOtherapeutics. To accelerate the development and clinical implementation of these compounds, several hurdles must be overcome, such as the lack of standardization in study design and the distance between preclinical investigations and potential clinical applications. The extant literature on neuroprotection utilizes a variety of animal and tissue models alongside ischemia models that range from global ischemia to focal occlusion on widely varying timescales. The lack of standardization impedes the comparisons and meta-analyses of different methods. As such, the guidelines for clinical trial design posited by Tymianski may be adapted to the preclinical evaluations of SUMOtherapeutics: study designs should emulate those of prior methodologically sound studies, subject heterogeneity should be minimized while treatment cohort size is maximized, and models should incorporate clinically relevant treatment windows (i.e., emphasis on co-treatment and post-treatment vs. pre-treatment) [107].

The difference between preclinical study design and putative clinical use case makes identifying knowledge gaps and pitfalls more difficult—small animal studies may provide translational utility if care is taken to replicate the clinical picture of ischemic stroke. Finally, promising neuroprotectants that were abandoned due to difficulties in translation may merit revisiting in forms that leverage the newer capabilities available today: in particular, perithrombectomy delivery at the time of recanalization and advances in targeted drug delivery to enhance blood–brain barrier penetration. Provided that these factors are considered, SUMOtherapeutics may represent the vanguard of an entirely new approach in the management of ischemic stroke.

## 4. Conclusions

The SUMO pathway represents a promising avenue for neuroprotection in the context of ischemic cerebral injury. The variety of druggable nodes and steadily increasing awareness of the scope of SUMO-mediated processes makes it an attractive target for therapeutic exploitation. SUMOtherapeutics for stroke may represent the first truly novel treatment paradigm in stroke since the discovery of tPA and the development of thrombectomy techniques. As such, a greater breadth and depth of preclinical inquiry with standardized methodologies and clinically congruent experimental design that utilizes recent advances in technology and technique is warranted.

## Data Availability

Data sharing not applicable.
